# FIB-4 Index and Diabetes Mellitus Are Associated with Chronic Kidney Disease in Japanese Patients with Non-Alcoholic Fatty Liver Disease

**DOI:** 10.3390/ijms21010171

**Published:** 2019-12-25

**Authors:** Yuya Seko, Kohta Yano, Aya Takahashi, Shinya Okishio, Seita Kataoka, Keiichiroh Okuda, Naoki Mizuno, Masashi Takemura, Hiroyoshi Taketani, Atsushi Umemura, Taichiro Nishikawa, Kanji Yamaguchi, Michihisa Moriguchi, Takeshi Okanoue, Yoshito Itoh

**Affiliations:** 1 Department of Molecular Gastroenterology and Hepatology, Kyoto Prefectural University of Medicine, Kyoto 6028566, Japan; 2 Department of Gastroenterology and Hepatology, Saiseikai Suita Hospital, Osaka 5640013, Japan

**Keywords:** nonalcoholic fatty liver disease, Type 2 diabetes, Hepatic fibrosis, PNPLA3

## Abstract

Non-alcoholic fatty liver disease (NAFLD) is associated with chronic kidney disease (CKD). The aim of this retrospective study was to determine the risk factors for progression of CKD in patients with biopsy-proven NAFLD including patatin-like phospholipase domain containing 3 (PNPLA3) polymorphism. A total of 344 patients with biopsy-proven NAFLD were enrolled consecutively in this study. Multivariate analysis identified males (odds ratio (OR) 5.46), age (per 1 year, OR 1.07), and FIB-4 index (≥1.30, OR 3.85) as factors associated with CKD. Of the 154 patients with a baseline estimated glomerular filtration rate (eGFR) ≥60 mL/min, 30 had a deterioration in CKD stage and 15 developed CKD after 3 years. Multivariate analysis identified diabetes mellitus (OR 2.44) as a risk factor for deterioration in CKD stage, while diabetes mellitus (OR 21.54) and baseline eGFR (per 1 mL/min OR 0.88) were risk factors for development of CKD. PNPLA3 did not affect the change in eGFR. In NAFLD patients, a high FIB-4 index was associated with CKD to increases in the index linked to reductions in eGFR. In order to prevent development of CKD, an appropriate therapy focusing on renal function is needed for NAFLD patients, especially those with diabetes.

## 1. Introduction

Non-alcoholic fatty liver disease (NAFLD) is the most common chronic liver disease in Japan and in all countries in the world and has a prevalence of 25–30% in the general adult population [1]. NAFLD covers a wide spectrum of diseases from non-alcoholic fatty liver (NAFL), which is usually benign, to non-alcoholic steatohepatitis (NASH), which can sometimes lead to liver cirrhosis or hepatocellular carcinoma (HCC) without significant alcohol consumption [2,3]. NAFLD also affects extra-hepatic disease such as type 2 diabetes mellitus (T2DM), cardiovascular disease, and chronic kidney disease (CKD) [4,5,6,7,8]. Of these diseases, there is growing evidence to support the concept that NAFLD affects the incidence of CKD. For example, a meta-analysis examined the association between CKD and NAFLD and showed that the risk of incident CKD in patients with NAFLD was significantly greater than in those without NAFLD (hazard ratio (HR) of 1.37). The analysis also showed that this risk increased with the severity of NAFLD [9]. Arase et al. reported that the annual incidence of CKD in Japanese patients with NAFLD was 1.2% and showed the risk of developing CKD was increased by five factors: low estimated glomerular filtration rate (eGFR), older age, T2DM, hypertension, and elevated gamma-glutamyltransferase (GGT) [10]. There is also evidence of the relationship between NAFLD and CKD from four long follow-up cohort studies [11,12,13,14]. However, in the majority of these earlier studies, the diagnosis of NAFLD was based on a non-invasive biochemistry index or ultrasonography, with the subjects detected mainly in health-check cohorts that were restricted to diabetic patients or men. It is well known that the non-synonymous single nucleotide polymorphisms (SNPs) of rs738409 in the patatin-like phospholipase domain containing 3 (PNPLA3) that encodes the p.I148M (isoleucine-to-methionine substitution at residue 148) is associated with the development and progression of NAFLD, NASH and hepatocellular carcinoma. There is therefore a paucity of longitudinal follow-up data of changes in renal function in outpatients with biopsy-proven NAFLD. Furthermore, whether improvement or deterioration in NAFLD and PNPLA3 polymorphism affect the progression or development of CKD remains unclear.

The objectives of this study were to clarify the prevalence of CKD and the risk factors for CKD in patients with biopsy-proven NAFLD, and to measure changes in liver function to determine the risk factors for deterioration and development of incident CKD including PNPLA3 polymorphism in a follow-up cohort.

## 2. Results

### 2.1. Patient Characteristics

**Figure 1:**
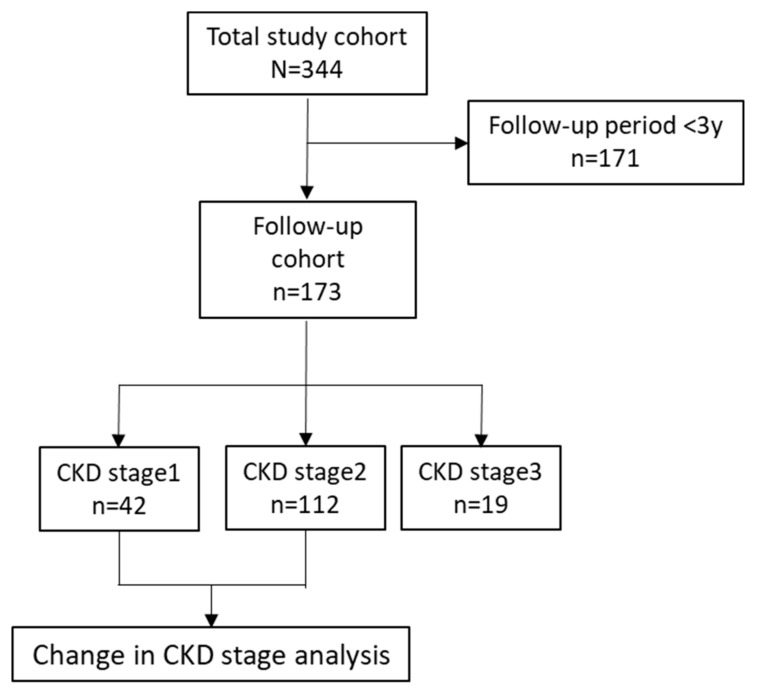
Flow diagram of the enrolled patients.

We enrolled 173 patients in the follow-up cohort after excluding 171 patients who had been followed-up for less than 3 years. A total of 154 patients with an eGFR >60 mL/min at baseline were analyzed for changes in CKD stage and incident CKD during the follow-up period (Figure 1). Table 1 summarizes the demographic profile and laboratory and histologic data of the total study and follow-up cohorts.

The total study cohort included 165 men (48.0%), median age of 57 years, with 255 patients (74.1%) diagnosed with NASH, 145 patients (42.2%) with hypertension, and 143 patients (41.6%) with T2DM. Sixteen of the patients in this cohort had cirrhosis. A FIB-4 index <1.30 was seen in 145 patients (42.2%) and a FIB-4 index ≥3.25 in 49 patients (14.2%). The 282 patients in total cohort, and 167 patients in follow-up cohort were analyzed PNPLA3 polymorphism.

### 2.2. Prevalence of Chronic Kidney Disease (CKD) and Associated Factors

Median eGFR was 78.7 mL/min in the total study cohort. Eighty-four patients (24.4%) were diagnosed as CKD stage 1, 225 patients (65.4%) as CKD stage 2, and 35 patients (10.2%) as CKD stage 3. Univariate analysis showed six parameters were associated significantly with CKD stage 3, with multivariate analysis identifying male (OR 5.46, *p* < 0.001), older age (OR 1.07, *p* = 0.003), and high FIB-4 index (≥1.30, OR 3.85, *p* = 0.036) (Table 2). The PNPLA3 polymorphism were not associated with CKD stage. The prevalence of CKD stage 3 in the subgroup with a FIB-4 index ≥1.30 was 15.6%, a value significantly greater than that in the FIB-4 index <1.30 subgroup (2.8%). The prevalence of patients with CKD stage 3 was not different according to PNPLA3 polymorphism.

### 2.3. Change in Renal Function and Risk Factors for Incident CKD

During the 3-year follow-up period, body mass index (BMI) and the results of liver function tests including those for aspartate aminotransferase (AST), alanine aminotransferase (ALT), and gamma-glutamyl transferase (GGT), total cholesterol, triglyceride, low-density lipoprotein cholesterol (LDL-C), and FIB-4 index were all significantly ameliorated. In contrast, eGFR decreased significantly from 80.0 mL/min to 75.5 mL/min (*p* < 0.001). In 42 patients with CKD stage 1 at baseline, 17 (40.5%) had a deterioration in renal function to stage 2, while 15 patients (13.4%) with stage 2 at baseline progressed to stage 3. Conversely, 17 patients (9.8%) had an improvement in their CKD stage during the follow-up period.

In the 154 patients with CKD stage 1 and 2 at baseline, 30 patients (19.5%) progressed to a worse CKD stage, while 15 patients (9.7%) had developed CKD by the end of the follow-up period. Table 3 shows a comparison of the baseline characteristics grouped according to changes in CKD stage and incidence of CKD. There were no significant differences between the two groups for the prevalence of NASH, sex, age, BMI, liver function including FIB-4 index, histological findings or PNPLA3 polymorphism. The prevalence of hypertension was significantly higher in the group that developed CKD than in the group that did not (*p* = 0.026). The prevalence of T2DM was significantly higher in the groups that had deterioration in CKD stage group or developed CKD (*p* = 0.026, *p* < 0.001, respectively). eGFR was significantly lower in the group that developed CKD compared with the group that did not. All the patients who developed CKD had stage 2 CKD at baseline. Liver histological severity and PNPLA3 polymorphism showed no association with change in CKD stage or incident CKD.

**Figure 2:**
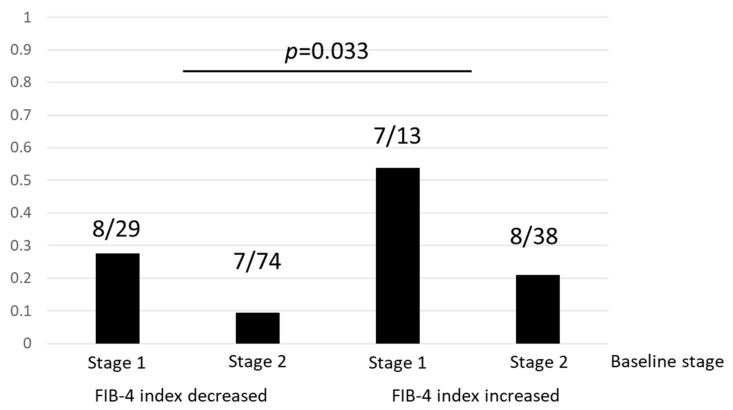
The prevalence of patients with a deterioration in chronic kidney disease stage according to a change in FIB-4 index.

**Figure 3:**
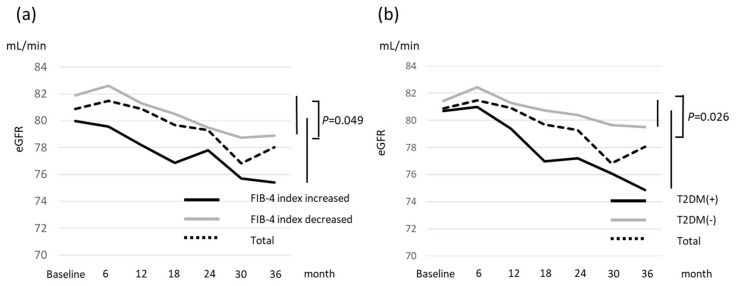
The median estimate glomerular filtration rate according to (**a**) change in FIB-4 index, and (**b**) presence of type 2 diabetes mellitus.

We performed multivariate analysis using sex, age, T2DM, hypertension, FIB-4 index at baseline, and eGFR at baseline as the covariables. This analysis identified T2DM (OR 2.44, *p* = 0.047) as a risk factor for a deterioration in CKD stage, while T2DM (OR 21.54, *p* = 0.005), and low eGFR at baseline (per 1mL/min, OR 0.88, *p* = 0.001) were risk factors for the development of CKD (Table 4). The prevalence of patients with a deterioration in CKD stage from 1 to 2, and 2 to 3 was 27.6% and 9.5%, respectively in the subgroup with a decreased FIB-4 index. The corresponding proportions in the subgroup with an increased FIB-4 index were 53.8% and 21.1%, respectively (Figure 2). Fifteen patients (29.4%) in the group with an increased FIB-4 index had a deterioration in CKD stage, a rate significantly greater than that observed in the group with a decreased FIB-4 index (14.6%) (*p* = 0.033) (Figure 2). There was no significant difference in eGFR at baseline in data stratified according to changes in the FIB-4 index or presence of T2DM. At the end of the follow-up period, eGFR in the group with a decreased FIB-4 index was significantly greater than that measured in the group with an increased index (*p* = 0.020). Patients with T2DM also had significantly lower eGFR than those without T2DM at the end of the follow-up period (*p* = 0.008). The change in eGFR in the group with an increased FIB-4 index was significantly greater than that in patients with a decrease in the index (*p* = 0.049) (Figure 3a). Median eGFR of patients with T2DM decreased from 80.7 mL/min at baseline to 74.9 mL/min at 3 years follow-up (*p* < 0.001), while patients without T2DM had a decrease from 81.4 mL/min to 79.5 mL/min (*p* = 0.004). This difference between the two groups was statistically significant (*p* = 0.026) (Figure 3b).

## 3. Discussion

This study investigated the prevalence of CKD and the association of the FIB-4 index with renal function in Japanese NAFLD patients. The FIB-4 index was not only associated with CKD, but also an increased index was a risk factor for deterioration in CKD stage. This study is the first report to clarify the correlation between changes in FIB-4 index and renal function in Japanese patients with NAFLD, based on the results of liver biopsies.

Of the study cohort, 10.2% had CKD, with data analysis showing a FIB-4 index ≥1.30 was an independent risk factor for development of CKD, in addition to male gender and older age. Previous studies in Japanese NAFLD cohorts have identified hypertension [10,15], eGFR <75 mL/min, ≥50 years old, T2DM, and a GGT ≥109 IU/L [10] as risk factors for CKD. The differences between these studies may reflect the characteristics of the cohorts such as the prevalence of males, NASH, and the diagnostic criteria for NAFLD. For example, the health check cohort included a high prevalence of men and younger subjects. In contrast, the hospital-based study included elderly subjects with a high prevalence of NASH and metabolic syndrome. Our data did not identify obesity as a risk factor for CKD. The influence of obesity on the development of CKD remains controversial. A large prospective observational cohort study in Korea reported that obesity was associated significantly with a 1.41-fold increase in the risk for adverse changes in renal function [16], whereas another large-scale cohort study in a CKD population with diabetes showed that the prevalence of metabolic syndrome, but not obesity, was associated significantly with CKD [17]. The influence of the metabolically healthy obese patients may be a reason for this discrepancy. Hashimoto et al. reported that the incidence of CKD with the metabolically healthy obesity phenotype was not higher than the incidence associated with the metabolically healthy non-obesity phenotype and lower than that associated with the metabolically abnormal non-obesity phenotype [18]. In our study, 116 of the 244 patients (47.5%) did not have metabolic syndrome, while 36 of the 102 non-obese patients (35.0%) were metabolically abnormal. These characteristics may have affected the associations we observed between obesity and CKD. In a large cohort study of Japanese subjects, higher age was identified as an independent risk factor of CKD [19]. The gender difference of risk in CKD was not clear according to previous studies. The higher risk of men in this study might be based on lifestyle habit including smoking which is known to be a risk factor of CKD. The severity of NAFLD has been reported to be associated with CKD in several cross-sectional and prospective studies [14,15,20,21,22]. The results of the current study including those of increases in the FIB-4 index ≥1.30 are consistent with previous studies.

In the follow-up cohort, median eGFR decreased by 4.5 mL/min over 3 years. Furthermore, 32 (20.8%) of patients had deterioration in CKD stage, with 15 (9.7%) developing CKD. The rate of eGFR decline in the Japanese general population has been reported to be 0.36 mL/min/year [23]. The presence of NAFLD may accelerate this decline. The change in eGFR in the FIB-4 index decreased or increased groups was –2.00 and –11.30 mL/min respectively. At the same time, the reduction of BMI and HbA1c appeared to have no effect on the change in eGFR. The pathophysiological links between FIB-4 index and renal function remain unclear. It is a well-accepted concept that both NAFLD and CKD are multisystem diseases and that there is interplay between the two diseases as a bidirectional cause and effect relationship. NAFLD per se and release of inflammatory, thrombogenic, oxidative, vasoactive, and fibrogenic mediators by adipose tissue lead to the development and progression of CKD [12,24,25]. Furthermore, NAFLD associated with T2DM exacerbates insulin resistance, causes atherogenic dyslipidemia, and activates the rennin-angiotensin system, all of which contribute to CKD [7]. Ectopic fat accumulation in the kidney also results in structural and functional adverse effects in the kidney [26,27]. Patients with an increased FIB-4 index would be expected to have systemic inflammatory status that is known to cause a deterioration in renal function.

In this study, the PNPLA3 polymorphism was not associated with renal function in both cohorts. Previous study by Sun, et al. reported that PNPLA3 GG allele were associated with renal function in NAFLD patients with normal ALT levels [28]. The difference between present and previous study might be based on the ALT levels of subjects in the study. The further large study is needed to confirm whether PNPLA3 is associated with renal function or not.

This study had several limitations. First, it was a retrospective study in a single center, with the number of subjects in the study not sufficient to confirm a conclusion. The change in BMI, AST, ALT, and HbA1c were not associated with change in eGFR in this study. A further longer prospective study using a larger number of subjects is, therefore, required to draw firm conclusions. Second, we did not collect information on proteinuria and also used a creatinine-based equation to estimate eGFR, a calculation that may not be accurate in patients with cirrhosis [29].

In conclusion, a FIB-4 index ≥1.30 was associated with CKD, while T2DM was a risk factor for a deterioration of renal function. Patients with a high FIB-4 index and those with increases in the index should receive careful consideration and monitoring of their renal function. In this regard, an appropriate therapeutic strategy for NAFLD patients with diabetes and CKD is needed.

## 4. Materials and Methods

### 4.1. Patients

A total of 344 Japanese patients diagnosed with NAFLD were enrolled in this retrospective study from January 2013 to April 2019. For inclusion in the study a liver biopsy finding of steatosis in ≥5% of hepatocytes were required, with patients excluded if they had been diagnosed with other liver diseases, including viral hepatitis, autoimmune hepatitis, and drug-induced liver disease. Patients consuming more than 20 g of alcohol per day and those with evidence of decompensated liver cirrhosis or hepatocellular carcinoma were also excluded. Of the 344 patients, 173 patients had been on medication for longer than 3 years and were enrolled in the follow-up cohort to examine changes in renal function and the relationship between liver function and renal function. All patients provided written informed consent and the study was conducted in accordance with the Declaration of Helsinki 2013. The study protocol was approved by the institution′s human research committee (approved on 13 September 2019, reference number; RBMR-G-129-7).

### 4.2. Laboratory and Clinical Parameters

Venous blood samples were collected in the morning after a 12-h overnight fast. Laboratory assays included blood cell counts and measurements of serum concentrations of aspartate aminotransferase (AST), alanine aminotransferase (ALT), GGT, total cholesterol, triglycerides, low-density lipoprotein cholesterol (LDL-C), high-density lipoprotein cholesterol (HDL-C), fasting plasma glucose (FPG), and type IV collagen 7s. These parameters were measured using standard clinical chemistry laboratory techniques. Hemoglobin A1c (HbA1c) was assayed using high-performance liquid chromatography and was expressed as National Glycohemoglobin Standardization Program (NGSP) units (%). Body mass index (BMI) was calculated as weight in kilograms/(height in meters) 2, with obesity defined as a BMI >25 kg/m^2^. T2DM was diagnosed according to the Report of the Expert Committee on the Diagnosis and Classification of Diabetes Mellitus or administration of anti T2DM agents. Patients with serum cholesterol concentrations >220 mg/dL or triglyceride concentrations >160 mg/dL or administration of anti-dyslipidemia agents were diagnosed with dyslipidemia. The FIB-4 index was calculated as follows: ([age (years) × AST (U/L)]/platelet count [109/L]) × (ALT [U/L])1/2. The following formulae for Japanese individuals were used to calculate eGFR according to gender [30]: for males, eGFR (mL/min/1.73 m ^2^) = 194 × [age] − 0.287 × [serum creatinine (mg/dL)] − 1.094; and for females, eGFR (mL/min/1.73 m^2^) = 194 × [age] − 0.287 × [serum creatinine (mg/dL)] − 1.094 × 0.739. CKD was defined as an estimated eGFR of <60 mL/min/1.73 m^2^ with its stages classified as follows: stage I, eGFR ≥ 90; stage II, 90 > eGFR ≥ 60, and stage III 60 > eGFR ≥ 30.

### 4.3. DNA Preparation and Single Nucleotide Polymorphism (SNP) Genotyping

Genomic DNA was extracted from blood samples using the DNeasy Blood and Tissue kit (Qiagen, Hilden, Germany). The SNP rs738409 was genotyped in each sample using TaqMan SNP genotyping assays (Applied Biosystems, Foster City, CA, USA). Of the 238 patients analyzed, 130 patients were determined to have PNPLA3 variants.

### 4.4. Liver Histology

**Figure 4:**
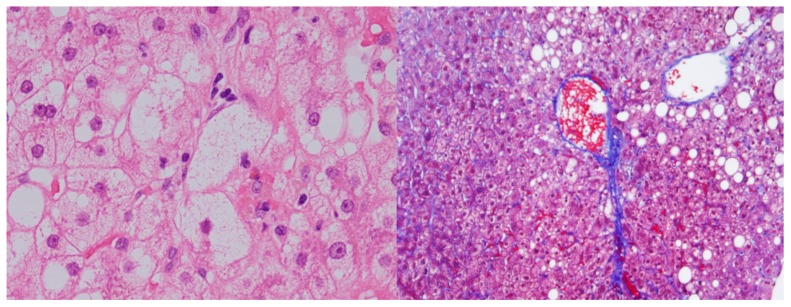
Liver biopsy findings of Hematoxylin and eosin (**left**) and Masson trichrome staining (**right**) in this study. (original magnifications: (**left**) ×400, (**right**) ×200).

All enrolled patients underwent a percutaneous liver biopsy under ultrasonic guidance. The liver specimens were embedded in paraffin and stained with hematoxylin and eosin, and Masson-trichrome (Figure 4). The specimens were evaluated by two hepatic pathologists who were blinded to the clinical findings. An adequate liver biopsy sample was defined as a specimen >1.5 cm long and/or having more than 11 portal tracts. NASH was defined as steatosis with lobular inflammation and ballooning degeneration, with or without Mallory–Denk bodies or fibrosis. Patients with liver biopsy specimens that showed simple steatosis or steatosis with non-specific inflammation were diagnosed with NAFL. Specimens with steatosis of <5, 5–33, >33–66, or >66% were scored as steatosis grades 0, 1, 2, and 3, respectively. For mild, moderate, and severe ballooning and inflammation (acinar and portal) the necroinflammatory grades were 1, 2, and 3, respectively. The severity of hepatic fibrosis (stage) was scored as: stage 1, zone 3 perisinusoidal fibrosis; stage 2, zone 3 perisinusoidal fibrosis with portal fibrosis; stage 3, zone 3 perisinusoidal fibrosis and portal fibrosis with bridging fibrosis; and stage 4, cirrhosis [31,32,33].

### 4.5. Statistical Analysis

The distribution of the subject’s characteristics was examined using the chi-square test or Mann–Whitney’s U test, as appropriate. We performed a logistic regression analysis to identify factors associated with CKD in the total cohort, after adjustment for sex, age, BMI, NAS steatosis, inflammation, and FIB-4 index (<1.30, ≥1.30). We also performed a logistic regression analysis in the follow-up cohort to evaluate risk factors for deterioration of CKD stage and development of CKD after adjustment for sex, age, T2DM, hypertension, FIB-4 index at baseline, and eGFR at baseline. All reported *p* values were two-sided with the significance level set at 0.05. The statistical comparisons were performed with SPSS software (SPSS Inc., Chicago, IL, USA).

## Figures and Tables

**Table 1:** Characteristics of the total and follow-up patient cohorts with non-alcoholic fatty liver disease.

Variable	Total Cohort (*n* = 344)	Follow-Up Cohort (*n* = 173)		
		Baseline	3 Years Later	*p*
NASH	255 (74.1%)	129 (74.6%)		
Hypertension	145 (42.2%)	79 (45.7%)		
T2DM	143 (41.6%)	73 (42.2%)		
Hyperlipidemia	209 (60.8%)	126 (72.8%)		
Sex, female	179 (52.0%)	88 (50.9%)		
Age, year	57 (22–84)	57 (25–79)	59 (28–82)	
BMI, kg/m^2^	27.0 (17.7–45.4)	26.8 (17.7–40.3)	26.7 (17.1–44.6)	0.003
Albumin, g/dL	4.5 (3.4–7.2)	4.5 (3.4–5.6)	4.4 (2.8–5.1)	<0.001
AST, IU/L	43 (13–650)	42 (12–208)	31 (12–212)	<0.001
ALT, IU/L	57 (10–615)	58 (10–263)	36 (11–248)	<0.001
GGT, IU/L	64 (13–716)	66 (14–355)	40 (12–565)	<0.001
Platelet count, × 10^3^/μL	217 (75–457)	210 (75–457)	214 (44–507)	0.004
Total cholesterol, mg/dL	199 (95–347)	203 (123–304)	194 (109–306)	0.009
Triglyceride, mg/dL	141 (10–923)	146 (10–739)	133 (46–574)	0.010
LDL-C, mg/dL	123 (36–435)	125 (48–435)	118 (54–213)	0.012
HDL-C, mg/dL	52 (16–107)	52 (25–107)	51 (22–95)	0.878
FPG, mg/dL	107 (61–374)	105 (61–325)	114 (72–349)	0.167
HbA1c, %	6.1 (4.7–11.0)	6.1 (5.0–11.0)	6.1 (5.1–11.1)	0.338
FIB-4 index	1.53 (0.26–11.06)	1.49 (0.27–7.84)	1.43 (0.30–13.64)	<0.001
eGFR, mL/min/1.73 m^2^	78.7 (37.2–165.4)	80.0 (40.9–154.9)	75.5 (31.0–142.4)	<0.001
CKD stage 1/2/3	84/225/35	42/112/19	37/107/29	<0.001
Type IV collagen 7s, ng/mL	5.0 (2.4–12.0)	4.9 (2.4–12.0)	4.8 (2.5–11.0)	0.002
Fibrosis stage (0/1/2/3/4)	120/109/61/38/16	65/50/34/13/11		
Steatosis (1/2/3)	99/185/60	37/107/29		
Inflammation (0/1/2/3)	14/200/110/20	5/99/55/14		
Ballooning (0/1/2)	105/144/95	46/67/60		
PNPLA3, CC/CG/GG	56/110/116	34/60/73		

Results are presented as *n* (%) for qualitative data or as median for quantitative data and within parenthesis are minimum to max values. Abbreviations: NASH, non-alcoholic steatohepatitis; T2DM, type 2 diabetes mellitus; BMI, body mass index; AST, aspartate aminotransferase; ALT, alanine aminotransferase; GGT, gamma-glutamyl transferase; LDL-C, low-density lipoprotein cholesterol; HDL-C, high-density lipoprotein cholesterol; FPG, fasting plasma glucose; eGFR, estimated glomerular filtration rate, CKD; chronic kidney disease; and PNPLA3, patatin-like phospholipase domain containing 3.

**Table 2:** Factors associated with chronic kidney disease in patients with non-alcoholic fatty liver disease in the total study cohort identified by multivariate analysis.

Variable	Multivariate Analysis	
	OR (95% CI) ^a^	*p* Value
Sex (male)	5.46 (2.35–12.70)	<0.001
Age (per 1 year)	1.07 (1.02–1.12)	0.003
BMI (per 1 kg/m^2^)	0.94 (0.85–1.05)	0.272
NAS steatosis (2, 3, 4)	0.84 (0.37–1.89)	0.665
NAS inflammation (3)	1.86 (0.52–6.68)	0.344
FIB-4 index (≥1.30)	3.85 (1.09–13.54)	0.036

The abbreviations are defined in Table 1. OR, Odds ratio; CI, confidence interval. ^a^ Estimated using logistic regression analysis.

**Table 3:** Demographic profiles and laboratory and histological data of patients, grouped according to the incidence of chronic kidney disease during the follow-up period.

Variable	Deterioration of CKD Stage			Incident CKD		
	Yes (*n* = 30)	No (*n* = 124)	*p*	Yes (*n* = 15)	No (*n* = 139)	*p*
NASH	20 (66.7%)	94 (75.8%)	0.355	11 (73.3%)	103 (74.1%)	1.000
Hypertension	17 (56.7%)	51 (41.1%)	0.153	11 (73.3%)	57 (41.0%)	0.026
Diabetes mellitus	19 (63.3%)	50 (40.3%)	0.026	14 (93.3%)	55 (39.6%)	<0.01
Hyperlipidemia	24 (80.0%)	88 (71.0%)	0.369	12 (80.0%)	100 (71.9%)	0.761
Sex, female	15 (50.0%)	69 (55.6%)	0.684	7 (46.7%)	77 (55.4%)	0.591
Age, year	57 (25–78)	55 (25–79)	0.290	57 (45–78)	55 (25–79)	0.115
BMI, kg/m^2^	27.4 (17.9–40.3)	26.9 (17.7–39.0)	0.804	26.2 (22.0–31.0)	27.1 (17.7–40.3)	0.715
Albumin, g/dL	4.5 (3.7–5.0)	4.5 (3.4–5.6)	0.790	4.5 (3.8–4.8)	4.5 (3.4–5.6)	0.648
AST, IU/L	42 (24–104)	42 (12–208)	0.786	45 (24–104)	42 (12–208)	0.853
ALT, IU/L	59.5 (31–150)	61 (10–263)	0.967	61 (31–138)	61 (10–263)	0.891
GGT, IU/L	66.5 (21–198)	67.5 (14–349)	0.814	80 (31–198)	66 (14–349)	0.304
Platelet count, × 10^3^/μL	201.5 (117–323)	221 (99–457)	0.256	168 (117–322)	221 (99–457)	0.071
Total cholesterol, mg/dL	212.5 (128–267)	202 (127–304)	0.266	216 (133–267)	202.5 (127–304)	0.538
Triglyceride, mg/dL	154.5 (50–739)	143.5 (10–584)	0.403	151 (68–739)	146 (10–584)	0.391
LDL-C, mg/dL	137 (92–177)	125 (66–435)	0.217	126 (92–169)	126 (66–435)	0.993
HDL-C, mg/dL	48.5 (30–84)	53.5 (25–107)	0.137	45 (30–72)	53 (25–107)	0.007
FPG, mg/dL	102.5 (72–212)	105.5 (61–325)	0.971	145 (72–212)	105 (61–325)	0.043
HbA1c, %	6.1 (5.3–8.9)	6.1 (5.0–11.0)	0.675	6.5 (5.9–8.9)	6.1 (5.0–11.0)	0.058
FIB-4 index	1.50 (0.40–4.44)	1.42 (0.27–7.84)	0.635	2.00 (0.61–4.44)	1.40 (0.27–7.84)	0.115
eGFR, mL/min/1.73 m^2^	88.7 (61.6–106.0)	80.8 (61.1–154.9)	0.967	66.6 (61.6–86.4)	81.4 (61.1–154.9)	<0.01
CKD stage 1/2	15/15	27/97	0.003	0/15	42/97	0.012
Type IV collagen 7s, ng/mL	4.5 (3.1–12.0)	5.0 (2.4–12.0)	0.792	4.5 (2.4–12.0)	4.9 (2.4–12.0)	0.601
Fibrosis stage (0/1/2/3/4)	13/6/5/5/1	46/37/28/5/8	0.099	6/4/3/1/1	55/39/30/9/8	1.000
Steatosis (1/2/3)	5/22/3	26/74/24	0.341	3/9/3	28/87/24	0.964
Inflammation (0/1/2/3)	0/18/10/2	5/71/41/7	0.733	0/9/4/2	5/80/47/7	0.502
Ballooning (0/1/2)	10/10/10	31/50/43	0.623	4/5/6	37/55/47	0.867
ΔBMI	−0.2 (−3.6–4.3)	−0.3 (−9.6–5.2)	0.841	0.1 (−3.6–1.3)	0.3 (−9.6–5.2)	0.528
ΔAST	−8.5 (−58–27)	−8 (−172–125)	0.575	–13 (−58–27)	–8 (−172–125)	0.798
ΔALT	−17.5 (−118–64)	−16 (−216–109)	0.743	–20 (−61–64)	–15 (−216–109)	0.961
ΔHbA1c	0.1 (−2.1–1.3)	0.1 (−3.2–2.6)	0.828	–0.4 (−2.1–1.3)	0.1 (−3.2–2.6)	0.142
ΔFIB-4 index	0.01 (−1.94–2.44)	−0.10 (−4.92–1.25)	0.106	0.10 (−1.94–2.44)	–0.10 (−4.92–1.25)	0.151
PNPLA3, CC/CG/GG	4/12/12	24/44/52	0.712	1/6/6	27/50/58	0.166

The abbreviations are defined in Table 1.

**Table 4:** Factors associated with deterioration of chronic kidney disease and incidence of chronic kidney disease in patients with non-alcoholic fatty liver disease in the follow-up cohort identified by multivariate analysis.

Variable	Deterioration of CKD Stage		Development of CKD	
	OR (95% CI) ^a^	*p* Value	OR (95% CI) ^a^	*p* Value
Sex		0.656		0.899
Age, year		0.464		0.535
T2DM	2.44 (1.01–5.91)	0.047	21.54 (2.50–185.33)	0.005
Hypertension		0.335		0.379
FIB-4 index at baseline		0.292		0.708
eGFR at baseline, mL/min		0.783	0.88 (0.81–0.95)	0.001

The abbreviations are defined in Table 1. OR, Odds ratio; CI, confidence interval; ^a^ Estimated using logistic regression analysis.

## Abbreviations

ALT	alanine aminotransferase
AST	aspartate aminotransferase
BMI	body mass index
CI	confidence interval
CKD	chronic kidney disease
eGFR	estimated glomerular filtration rate
FPG	fasting plasma glucose
GGT	γ glutamyl transpeptidase
HbA1c	hemoglobin A1c
HDL-C	high-density lipoprotein cholesterol
LDL-C	low-density lipoprotein cholesterol
NAFLD	nonalcoholic fatty liver disease
NASH	nonalcoholic steatohepatitis
OR	odds ratio
T2DM	type 2 diabetes mellitus
PNPLA3	patatin-like phospholipase domain containing 3
